# The Committee on Voluntary Hospitals

**Published:** 1921-01-22

**Authors:** 


### THE COMMITTEE ON VOLUNTARY HOSPITALS.

At last have been made public the names of some members of the Committee appointed by the Minister of Health to consider the financial position of voluntary hospitals. Viscount Cave is the Chairman; he was for many years one of the most brilliant advocates practising in the courts, Member of Parliament for the Richmond division of Surrey, and, before he was appointed a Lord of Appeal, was for a period Home Secretary. His legal mind, combined with an experience gathered from a long and varied public career, should be of great service to the hospitals in the present juncture. The other members—Sir Clarendon Hyde, also a lawyer and a member of the firm of S. Pearson and Son, contractors, of which Lord Cowdray is head; Mr. R. C. Norman, late Chairman of L.C.C., with his special knowledge of the local government systems of the Metropolis; and Mr. Vernon Hartshorn, a Labour Member of Parliament who served on the Coal Controller's Advisory Committee—should prove useful committee men. Two other members are still to be appointed—one a representative of Scotland and one a chartered accountant.

It is to be hoped that the Committee will get to work at once (there has been too much delay as it is) and will quickly, in the words of the reference, "make recommendations as to any action that should be taken to assist" voluntary hospitals. Mr. L. G. Brock, C.B., of the Ministry of Health, is the Secretary, and all offers of evidence, of which, no doubt, there will be a number, should be sent to him.

In the meantime, we hope that the Press and hospital people generally will leave hospital politics, as far as possible, alone. In this connection we rather regret the recent duel in the Correspondence Column of *The Times* between Sir Ernest Hatch and Sir Arthur Stanley, with a letter thrown in as a makeweight by Mr. Glenton-Kerr. Hospitals certainly cannot live without money, and why the Lord Mayor's modest letter of appeal on behalf of St. Thomas's should have created such a stir we do not know, nor why at this time anybody wishes to argue about the profitableness of receiving wounded soldiers in voluntary hospitals.

There is clearly no reason why hospitals should not continue to help themselves. Lord Cave's Committee is certainly not going to produce a scheme which will provide all the money needful. Especially interesting at the present moment is the scheme suggested by Dr. Gordon Dill for Brighton, which is detailed on another page. If we remember rightly, it was Mr. Gilbert Panter, of the Great Northern Hospital, who first mapped out in these columns a provident plan of this kind (see The Hospital, July 24, 1920, p. 431), and Mr. Coles, of King's College Hospital, has made some useful contributions to its consideration. Sir Arthur Stanley says that he and Sir Napier Burnett have been looking into the subject for some time past, and he is convinced that now the London hospitals have come together and have formed a strong committee under the auspices of the British Hospitals Association, such a scheme, if approved, could be launched by the committee at an early date. A small weekly or monthly contribution from every household in London, Sir Arthur Stanley thinks, carrying with it the assurance of such hospital treatment as might be necessary for any members of that household, would readily meet the financial needs of the hospitals.

Such an important and apparently workable method of finance is one which should be brought to the notice of the Committee of Enquiry, and we hope that Sir Arthur Stanley will have the scheme worked out in all its details in time for this purpose.

But this is but one of the matters which will engage the attention of the Committee of Inquiry. Without in any way suggesting what the findings will be, we can, not improperly, contemplate the present temper of the taxpayer. It is not a time for any considerable increase in the way of costly social schemes which will rely largely upon rates and taxes for their subsistence. Therefore, reasonable ideas which will gather in the nimble sixpence will be more likely to secure ready consideration than schemes entailing the loading of further millions upon the breaking backs of the comparative few.

